# Poly[[diaqua­manganese(II)]-bis­(μ-4-fluoro­benzoato-κ^2^ *O:O*′)]

**DOI:** 10.1107/S1600536811021921

**Published:** 2011-06-30

**Authors:** Hacali Necefoğlu, Füreya Elif Özbek, Vijdan Öztürk, Barış Tercan, Tuncer Hökelek

**Affiliations:** a Kafkas University, Department of Chemistry, 36100 Kars, Turkey; b Karabük University, Department of Physics, 78050, Karabük, Turkey; c Hacettepe University, Department of Physics, 06800 Beytepe, Ankara, Turkey

## Abstract

In the crystal structure of the title complex, [Mn(C_7_H_4_FO_2_)_2_(H_2_O)_2_]_*n*_, the Mn^II^ atom is located on an inversion centre. It is coordinated by two water mol­ecules in the apical directions and four 4-fluoro­benzoate (PFB) anions, bridging the symmetry related Mn atoms in the basal plane to form an infinite two-dimensional polymeric structure parallel to (100). The four O atoms of the PFB anions around the Mn^II^ atom form a slightly distorted square-planar arrangement, while the slightly distorted octa­hedral coordination is completed by the two O atoms of the water mol­ecules. The dihedral angle between the carboxyl­ate group and the adjacent benzene ring is 27.29 (16)°. The O—H⋯O hydrogen bonds further connect the manganese-carboxyl­ate units. π–π contacts between the benzene rings [centroid-centroid distance = 3.6894 (15) Å] further stabilize the crystal structure.

## Related literature

For literature on niacin, see: Krishnamachari (1974 ▶). For infomation on the nicotinic acid derivative *N*,*N*-diethyl­nicotinamide, see: Bigoli *et al.* (1972 ▶). For related structures, see: Hökelek *et al.* (2008 ▶, 2009 ▶); Hökelek & Necefoğlu (2007 ▶). For bond-length data, see: Allen *et al.* (1987 ▶).
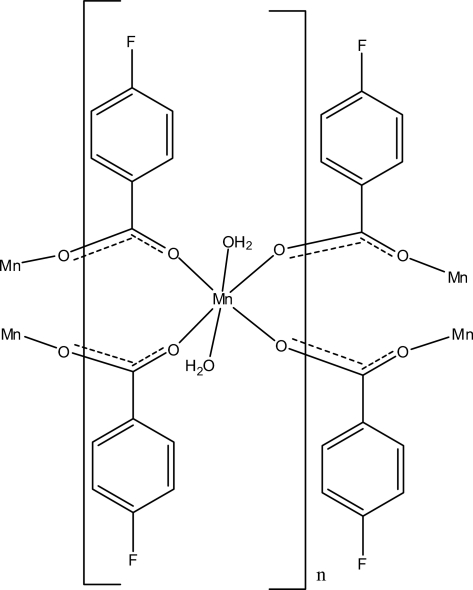


## Experimental

### 

#### Crystal data

[Mn(C_7_H_4_FO_2_)_2_(H_2_O)_2_]
*M* *_r_* = 369.18
Monoclinic, 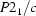

*a* = 14.5065 (6) Å
*b* = 6.6107 (3) Å
*c* = 7.3708 (3) Å
β = 98.179 (2)°
*V* = 699.66 (5) Å^3^
*Z* = 2
Mo *K*α radiation
μ = 1.00 mm^−1^
*T* = 100 K
0.34 × 0.27 × 0.24 mm

#### Data collection

Bruker Kappa APEXII CCD area-detector diffractometer
Absorption correction: multi-scan (*SADABS*; Bruker, 2005 ▶) *T*_min_ = 0.728, *T*_max_ = 0.786
11656 measured reflections
1758 independent reflections
1720 reflections with *I* > 2σ(*I*)
*R*_int_ = 0.029

#### Refinement

*R*[*F* ^2^ > 2σ(*F* ^2^)] = 0.039
*wR*(*F* ^2^) = 0.111
*S* = 1.28
1758 reflections
114 parameters
H atoms treated by a mixture of independent and constrained refinement
Δρ_max_ = 1.24 e Å^−3^
Δρ_min_ = −0.45 e Å^−3^

### 

Data collection: *APEX2* (Bruker, 2007 ▶); cell refinement: *SAINT* (Bruker, 2007 ▶); data reduction: *SAINT*; program(s) used to solve structure: *SHELXS97* (Sheldrick, 2008 ▶); program(s) used to refine structure: *SHELXL97* (Sheldrick, 2008 ▶); molecular graphics: *ORTEP-3 for Windows* (Farrugia, 1997 ▶); software used to prepare material for publication: *WinGX* publication routines (Farrugia, 1999 ▶) and *PLATON* (Spek, 2009 ▶).

## Supplementary Material

Crystal structure: contains datablock(s) I, global. DOI: 10.1107/S1600536811021921/su2282sup1.cif

Structure factors: contains datablock(s) I. DOI: 10.1107/S1600536811021921/su2282Isup2.hkl

Additional supplementary materials:  crystallographic information; 3D view; checkCIF report

## Figures and Tables

**Table 1:** Hydrogen-bond geometry (Å, °)

*D*—H⋯*A*	*D*—H	H⋯*A*	*D*⋯*A*	*D*—H⋯*A*
O3—H32⋯O2^i^	0.79 (4)	2.51 (4)	3.039 (3)	125 (4)
O3—H32⋯O1^i^	0.79 (4)	2.18 (4)	2.935 (3)	158 (4)

Symmetry code: (i) 
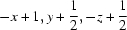
.

**Fig. 1.**
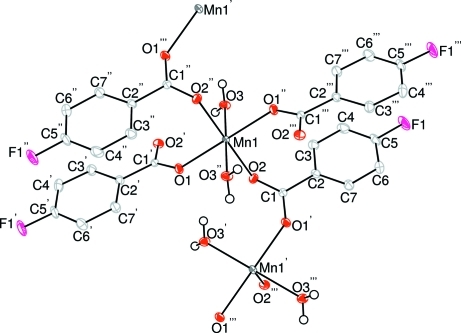
The molecular structure of the title molecule with the atom-numbering scheme. Displacement ellipsoids are drawn at the 50% probability level [symmetry codes: (') -x, y + 1/2, 1/2 - z, ('') -x, -y, -z, (''') x, 1/2 - y, 1/2 + z]. Hydrogen atoms, except those of the water molecules, have been omitted for clarity.

**Fig. 2.**
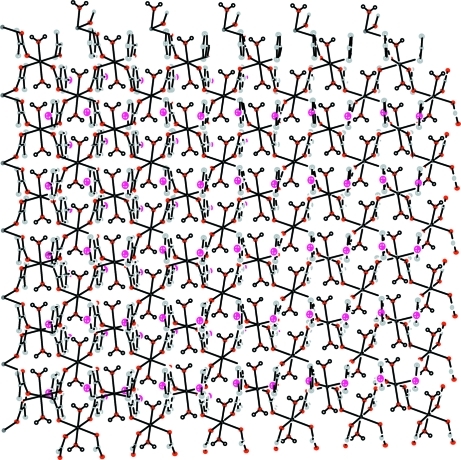
A view along the a-axis of the crystal packing of the title compound [c-axis horizontal; b-axis vertical]. Hydrogen atoms, except those of the water molecules (violet balls), have been omitted for clarity.

## supplementary crystallographic information

### Comment

As a part of our ongoing investigations of transition metal complexes of nicotinamide (NA), one form of niacin (Krishnamachari, 1974), and/or the nicotinic acid derivative *N*,*N*-diethylnicotinamide (DENA), an important respiratory stimulant (Bigoli *et al.*, 1972), the title compound was synthesized and its crystal structure is reported herein.

In the title two-dimensional polymeric structure the Mn^II^ atom is located on a centre of invesion, and surrounded by four 4-fluorobenzoate (PFB) anions and two water molecules (Fig. 1). The PFB anions bridge the symmetry related Mn atoms. The four O atoms [O1, O2, O1'' and O2'', symmetry code: ('') -x, -y, -z] in the equatorial plane around the Mn atom form a slightly distorted square-planar arrangement, while the slightly distorted octahedral coordination is completed by the symmetry related O atoms of the coordinated water molecules (O3 and O3'') in the axial positions (Fig. 1).

The near equalities of the C1—O1 [1.257 (3) Å] and C1—O2 [1.268 (3) Å] bonds in the carboxylate group indicate a delocalized bonding arrangement, rather than localized single and double bonds, and may be compared with the corresponding distances: 1.263 (2), 1.279 (2), 1.263 (2) and 1.278 (2) Å in {[Mn(C_11_H_14_NO_2_)_2_(H_2_O)_3_].2(H_2_O)}_n_, (II) (Hökelek *et al.*, 2009), 1.256 (6) and 1.245 (6) Å in [Mn(DENA)_2_(C_7_H_4_ClO_2_)_2_(H_2_O)_2_], (III) (Hökelek *et al.*, 2008) and 1.265 (6) and 1.275 (6) Å in [Mn(C_9_H_10_NO_2_)_2_(H_2_O)_4_].2(H_2_O), (IV) (Hökelek & Necefoğlu, 2007).

The Mn—O bond lengths are in the range of 2.1489 (17) - 2.1988 (19) Å, and are close to standard values (Allen *et al.*, 1987) with an average Mn-O bond length of 2.1735 (18) Å. The Mn atom is displaced out of the least-square plane of the carboxylate group (O1/C1/O2) by -1.5976 (1) Å. The dihedral angle between the planar carboxylate group and the adjacent benzene ring A (C2—C7) is 27.29 (16)°.

In the crystal structure, (Fig. 2), intermolecular O—H···O hydrogen bonds (Table 1) link the manganese-carboxylate units, and may be effective in the stabilization of the structure. The π···π contacts between the benzene rings, Cg1—Cg1^i^ [symmetry code: (i) x, 1/2 - y, z - 1/2, where Cg1 is the centroid of the ring A (C2—C7)] may further stabilize the structure, with a centroid-centroid distance of 3.6894 (15) Å.

### Experimental

The title compound was prepared by the reaction of MnSO_4_.H_2_O (0.85 g, 5 mmol) in H_2_O (100 ml) and isonicotinamide (1.22 g, 10 mmol) in H_2_O (50 ml) with sodium 4-fluorobenzoate (1.62 g, 10 mmol) in H_2_O (50 ml) at room temperature. The mixture was filtered and set aside to crystallize at ambient temperature for two weeks, giving blue single crystals.

### Refinement

Atoms H31 and H32 (for H_2_O) were located in a difference Fourier map and were freely refined. The C-bound H-atoms were positioned geometrically with C—H = 0.95 Å for aromatic H-atoms, and constrained to ride on their parent atoms, with *U*_iso_(H) = *1.2U*_eq_(C).

### Crystal data

**Table:**

[Mn(C_7_H_4_FO_2_)_2_(H_2_O)_2_]	*F*(000) = 374
*M**_r_* = 369.18	*D*_x_ = 1.752 Mg m^−^^3^
Monoclinic, *P*2_1_/*c*	Mo *K*α radiation, λ = 0.71073 Å
Hall symbol: -P 2ybc	Cell parameters from 9752 reflections
*a* = 14.5065 (6) Å	θ = 2.8–28.5°
*b* = 6.6107 (3) Å	µ = 1.00 mm^−^^1^
*c* = 7.3708 (3) Å	*T* = 100 K
β = 98.179 (2)°	Block, blue
*V* = 699.66 (5) Å^3^	0.34 × 0.27 × 0.24 mm
*Z* = 2	

### Data collection

**Table:**

Bruker Kappa APEXII CCD area-detector diffractometer	1758 independent reflections
Radiation source: fine-focus sealed tube	1720 reflections with *I* > 2σ(*I*)
graphite	*R*_int_ = 0.029
φ and ω scans	θ_max_ = 28.5°, θ_min_ = 2.8°
Absorption correction: multi-scan (*SADABS*; Bruker, 2005)	*h* = −19→19
*T*_min_ = 0.728, *T*_max_ = 0.786	*k* = −8→7
11656 measured reflections	*l* = −9→9

### Refinement

**Table:**

Refinement on *F*^2^	Primary atom site location: structure-invariant direct methods
Least-squares matrix: full	Secondary atom site location: difference Fourier map
*R*[*F*^2^ > 2σ(*F*^2^)] = 0.039	Hydrogen site location: inferred from neighbouring sites
*wR*(*F*^2^) = 0.111	H atoms treated by a mixture of independent and constrained refinement
*S* = 1.28	*w* = 1/[σ^2^(*F*_o_^2^) + (0.031*P*)^2^ + 1.8232*P*] where *P* = (*F*_o_^2^ + 2*F*_c_^2^)/3
1758 reflections	(Δ/σ)_max_ < 0.001
114 parameters	Δρ_max_ = 1.24 e Å^−^^3^
0 restraints	Δρ_min_ = −0.45 e Å^−^^3^

### Special details

**Table:**

Geometry. All esds (except the esd in the dihedral angle between two l.s. planes) are estimated using the full covariance matrix. The cell esds are taken into account individually in the estimation of esds in distances, angles and torsion angles; correlations between esds in cell parameters are only used when they are defined by crystal symmetry. An approximate (isotropic) treatment of cell esds is used for estimating esds involving l.s. planes.
Refinement. Refinement of F^2^ against ALL reflections. The weighted R-factor wR and goodness of fit S are based on F^2^, conventional R-factors R are based on F, with F set to zero for negative F^2^. The threshold expression of F^2^ > 2sigma(F^2^) is used only for calculating R-factors(gt) etc. and is not relevant to the choice of reflections for refinement. R-factors based on F^2^ are statistically about twice as large as those based on F, and R- factors based on ALL data will be even larger.

### Fractional atomic coordinates and isotropic or equivalent isotropic displacement parameters (Å^2^)

**Table:**

	*x*	*y*	*z*	*U*_iso_*/*U*_eq_	
Mn1	0.5000	1.0000	0.5000	0.00934 (15)	
O1	0.39762 (12)	1.0833 (3)	0.2723 (2)	0.0133 (4)	
O2	0.58287 (12)	0.9092 (3)	0.2904 (2)	0.0133 (4)	
O3	0.57162 (13)	1.2939 (3)	0.5150 (3)	0.0146 (4)	
H31	0.575 (3)	1.370 (6)	0.604 (6)	0.027 (10)*	
H32	0.566 (3)	1.361 (6)	0.425 (6)	0.031 (10)*	
F1	0.99509 (11)	0.7768 (3)	0.6699 (2)	0.0279 (4)	
C1	0.63126 (16)	0.7488 (4)	0.2983 (3)	0.0099 (4)	
C2	0.72835 (17)	0.7562 (4)	0.3993 (3)	0.0123 (5)	
C3	0.76860 (18)	0.9415 (4)	0.4534 (4)	0.0169 (5)	
H3	0.7340	1.0629	0.4285	0.020*	
C4	0.85953 (19)	0.9492 (4)	0.5439 (4)	0.0207 (5)	
H4	0.8885	1.0748	0.5792	0.025*	
C5	0.90623 (17)	0.7696 (5)	0.5807 (4)	0.0184 (5)	
C6	0.86858 (19)	0.5840 (4)	0.5321 (4)	0.0200 (5)	
H6	0.9030	0.4633	0.5614	0.024*	
C7	0.77816 (18)	0.5780 (4)	0.4383 (4)	0.0168 (5)	
H7	0.7505	0.4517	0.4008	0.020*	

### Atomic displacement parameters (Å^2^)

**Table:**

	*U*^11^	*U*^22^	*U*^33^	*U*^12^	*U*^13^	*U*^23^
Mn1	0.0082 (2)	0.0104 (3)	0.0089 (2)	0.00104 (17)	−0.00066 (17)	−0.00106 (17)
O1	0.0120 (8)	0.0117 (8)	0.0150 (8)	0.0013 (6)	−0.0025 (6)	0.0011 (6)
O2	0.0145 (8)	0.0125 (8)	0.0131 (8)	0.0030 (7)	0.0025 (6)	0.0003 (6)
O3	0.0200 (9)	0.0128 (8)	0.0106 (8)	−0.0009 (7)	0.0010 (7)	0.0003 (7)
F1	0.0112 (7)	0.0423 (11)	0.0272 (9)	−0.0031 (7)	−0.0071 (6)	−0.0018 (8)
C1	0.0092 (10)	0.0115 (10)	0.0091 (10)	−0.0004 (8)	0.0024 (8)	0.0008 (8)
C2	0.0117 (10)	0.0154 (11)	0.0097 (10)	−0.0011 (8)	0.0010 (8)	−0.0007 (8)
C3	0.0163 (12)	0.0152 (12)	0.0185 (12)	−0.0015 (9)	−0.0002 (9)	−0.0012 (9)
C4	0.0176 (12)	0.0210 (13)	0.0226 (13)	−0.0073 (10)	−0.0003 (10)	−0.0053 (10)
C5	0.0083 (10)	0.0314 (15)	0.0147 (11)	−0.0023 (10)	−0.0014 (9)	−0.0008 (10)
C6	0.0153 (12)	0.0219 (13)	0.0216 (13)	0.0046 (10)	−0.0016 (10)	0.0013 (10)
C7	0.0154 (11)	0.0153 (12)	0.0184 (12)	0.0002 (9)	−0.0016 (9)	−0.0010 (9)

### Geometric parameters (Å, °)

**Table:**

Mn1—O1	2.1489 (17)	C1—C2	1.498 (3)
Mn1—O1^i^	2.1489 (17)	C2—C3	1.391 (3)
Mn1—O2	2.1728 (17)	C2—C7	1.390 (3)
Mn1—O2^i^	2.1728 (17)	C3—H3	0.9500
Mn1—O3	2.1988 (19)	C4—C3	1.392 (4)
Mn1—O3^i^	2.1988 (19)	C4—H4	0.9500
O1—C1^ii^	1.257 (3)	C5—C4	1.375 (4)
O2—C1	1.268 (3)	C6—C5	1.370 (4)
O3—H31	0.82 (4)	C6—H6	0.9500
O3—H32	0.79 (4)	C7—C6	1.394 (4)
F1—C5	1.362 (3)	C7—H7	0.9500
C1—O1^iii^	1.257 (3)		
			
O1—Mn1—O1^i^	180.0	O1^iii^—C1—C2	117.9 (2)
O1—Mn1—O2	84.62 (7)	O2—C1—C2	118.1 (2)
O1^i^—Mn1—O2	95.38 (7)	C3—C2—C1	119.9 (2)
O1—Mn1—O2^i^	95.38 (7)	C7—C2—C1	120.0 (2)
O1^i^—Mn1—O2^i^	84.62 (7)	C7—C2—C3	120.2 (2)
O1—Mn1—O3	94.69 (7)	C2—C3—C4	120.0 (2)
O1^i^—Mn1—O3	85.31 (7)	C2—C3—H3	120.0
O1—Mn1—O3^i^	85.31 (7)	C4—C3—H3	120.0
O1^i^—Mn1—O3^i^	94.69 (7)	C3—C4—H4	121.0
O2—Mn1—O2^i^	180.0	C5—C4—C3	118.0 (2)
O2—Mn1—O3	88.55 (7)	C5—C4—H4	121.0
O2^i^—Mn1—O3	91.45 (7)	F1—C5—C4	118.1 (2)
O2—Mn1—O3^i^	91.45 (7)	F1—C5—C6	118.2 (2)
O2^i^—Mn1—O3^i^	88.55 (7)	C6—C5—C4	123.7 (2)
O3—Mn1—O3^i^	180.00 (10)	C5—C6—C7	117.9 (2)
C1^ii^—O1—Mn1	134.33 (16)	C5—C6—H6	121.1
C1—O2—Mn1	123.85 (15)	C7—C6—H6	121.1
Mn1—O3—H31	124 (3)	C2—C7—C6	120.2 (2)
Mn1—O3—H32	118 (3)	C2—C7—H7	119.9
H31—O3—H32	108 (4)	C6—C7—H7	119.9
O1^iii^—C1—O2	124.0 (2)		
			
O2—Mn1—O1—C1^ii^	−106.1 (2)	O2—C1—C2—C7	169.1 (2)
O2^i^—Mn1—O1—C1^ii^	73.9 (2)	C1—C2—C3—C4	−178.6 (2)
O3—Mn1—O1—C1^ii^	−18.0 (2)	C7—C2—C3—C4	1.1 (4)
O3^i^—Mn1—O1—C1^ii^	162.0 (2)	C1—C2—C7—C6	179.9 (2)
O1^i^—Mn1—O2—C1	41.54 (19)	C3—C2—C7—C6	0.2 (4)
O3—Mn1—O2—C1	126.69 (19)	C5—C4—C3—C2	−1.4 (4)
O3^i^—Mn1—O2—C1	−53.31 (19)	F1—C5—C4—C3	−179.8 (2)
Mn1—O2—C1—O1^iii^	93.1 (3)	C6—C5—C4—C3	0.5 (4)
Mn1—O2—C1—C2	−86.2 (2)	C7—C6—C5—F1	−179.0 (2)
O1^iii^—C1—C2—C3	169.5 (2)	C7—C6—C5—C4	0.7 (4)
O1^iii^—C1—C2—C7	−10.3 (3)	C2—C7—C6—C5	−1.1 (4)
O2—C1—C2—C3	−11.2 (3)		

Symmetry codes: (i) −*x*+1, −*y*+2, −*z*+1; (ii) −*x*+1, *y*+1/2, −*z*+1/2; (iii) −*x*+1, *y*−1/2, −*z*+1/2.

### Hydrogen-bond geometry (Å, °)

**Table:**

*D*—H···*A*	*D*—H	H···*A*	*D*···*A*	*D*—H···*A*
O3—H32···O2^ii^	0.79 (4)	2.51 (4)	3.039 (3)	125 (4)
O3—H32···O1^ii^	0.79 (4)	2.18 (4)	2.935 (3)	158 (4)

Symmetry codes: (ii) −*x*+1, *y*+1/2, −*z*+1/2.

## References

[bb1] Allen, F. H., Kennard, O., Watson, D. G., Brammer, L., Orpen, A. G. & Taylor, R. (1987). *J. Chem. Soc., Perkin Trans. 2*, pp. S1–19.

[bb2] Bigoli, F., Braibanti, A., Pellinghelli, M. A. & Tiripicchio, A. (1972). *Acta Cryst.* B**28**, 962–966.

[bb3] Bruker (2005). *SADABS* Bruker AXS Inc. Madison, Wisconsin, USA.

[bb4] Bruker (2007). *APEX2* and *SAINT* Bruker AXS Inc. Madison, Wisconsin, USA.

[bb5] Farrugia, L. J. (1997). *J. Appl. Cryst.* **30**, 565.

[bb6] Farrugia, L. J. (1999). *J. Appl. Cryst.* **32**, 837–838.

[bb7] Hökelek, T., Çaylak, N. & Necefoğlu, H. (2008). *Acta Cryst.* E**64**, m505–m506. 10.1107/S1600536808005540 PMC2960864 21201885

[bb8] Hökelek, T., Dal, H., Tercan, B., Aybirdi, Ö. & Necefoğlu, H. (2009). *Acta Cryst.* E**65**, m747–m748. 10.1107/S1600536809021060 PMC2969307 21582685

[bb9] Hökelek, T. & Necefoğlu, H. (2007). *Acta Cryst.* E**63**, m821–m823.

[bb10] Krishnamachari, K. A. V. R. (1974). *Am. J. Clin. Nutr.* **27**, 108–111. 10.1093/ajcn/27.2.108 4812927

[bb11] Sheldrick, G. M. (2008). *Acta Cryst.* A**64**, 112–122. 10.1107/S0108767307043930 18156677

[bb12] Spek, A. L. (2009). *Acta Cryst.* D**65**, 148–155. 10.1107/S090744490804362X PMC2631630 19171970

